# Species-Dependent Aggregation of Oral Bacteria Under Simulated Microgravity

**DOI:** 10.3390/life16091402

**Published:** 2026-08-25

**Authors:** Ryosuke Hanai, Makoto Hirohata, Yoshihiko Sugita, Hisataka Kondo, Yoshikazu Naiki, Kiyoshi Nishikawa, Mayu Hida, Yoshiaki Hasegawa, Ken Miyazawa, Hatsuhiko Maeda

**Affiliations:** 1 Department of Orthodontics, School of Dentistry, Aichi Gakuin University, 2-11 Suemori-dori, Chikusa-ku, Nagoya 464-8651, Japan; 2 Department of Microbiology, School of Dentistry, Aichi Gakuin University, 1-100 Kusumoto-cho, Chikusa-ku, Nagoya 464-8650, Japan; 3 Department of Oral Pathology/Forensic Odontology, School of Dentistry, Aichi Gakuin University, 1-100 Kusumoto-cho, Chikusa-ku, Nagoya 464-8650, Japan; 4 Aichi Gakuin University Space Research Center, 1-100 Kusumoto-cho, Chikusa-ku, Nagoya 464-8650, Japan; 5 Junior College, Aichi Gakuin University, Nagoya 464-8651, Japan

**Keywords:** simulated microgravity, oral bacteria, biofilm formation, streptococcus mutans, extracellular polysaccharides

## Abstract

**Objective:** Simulated microgravity (SMG) alters fluid dynamics and may influence microbial aggregation. However, its effects on oral bacteria remain unclear. This study investigated the effects of SMG on the growth and aggregation of representative oral bacteria. **Methods:** Four facultative oral bacteria representing distinct ecological and adhesive properties—*Staphylococcus aureus* (opportunistic pathogen), *Streptococcus mutans* (glucan-producing cariogenic bacterium), *Streptococcus salivarius* (commensal fructan-producing bacterium), and *Streptococcus sanguinis* (early colonizer)—together with the periodontal pathogen *Porphyromonas gingivalis* were cultured under SMG (10^−3^G) or normal gravity (NG). Streptococci were grown with or without sucrose to evaluate the role of extracellular polysaccharides. Growth was assessed by optical density, and aggregation was quantified by image analysis. **Results:** SMG induced aggregation in a species-dependent manner. *S. mutans* and *S. salivarius* formed spherical aggregates under SMG, whereas little or no aggregate formation was observed in *S. aureus*, *S. sanguinis*, or *P. gingivalis*. Aggregation of *S. mutans* was markedly reduced without sucrose, indicating dependence on glucan-mediated adhesion. *S. salivarius* also showed reduced aggregation under sucrose-free conditions, although limited aggregation persisted. Growth kinetics were virtually unaffected by SMG. **Conclusions:** SMG promotes aggregation of selected oral bacteria through species-specific mechanisms. These findings suggest that microgravity may alter the architecture of oral microbial communities by enhancing particular intercellular adhesion pathways.

## 1. Introduction

Microgravity environments, such as those encountered during spaceflight, suppress sedimentation and convective flow, resulting in a low-shear fluid environment that can significantly alter microbial behavior, including growth, stress responses, and biofilm formation [1,2].

Because true microgravity experiments are limited, simulated microgravity (SMG) systems such as clinostats are widely used to reproduce key physical aspects of spaceflight conditions on Earth [2,3]. Although SMG cannot fully replicate space environments, it provides a useful model for investigating gravity-dependent microbial physiology. Previous studies have shown that microgravity can influence bacterial biofilm formation, virulence factor expression, stress adaptation, and cellular physiology [4]. For example, Orsini et al. investigated the effects of SMG on the physiology and global gene expression of *Streptococcus mutans* and reported changes in gene expression related to carbohydrate metabolism, translation, and stress responses [5]. Importantly, they also observed a distinct aggregation phenotype under SMG, with *S. mutans* forming rounder and more compact cellular aggregates than those formed under normal gravity conditions. These findings suggest that altered gravitational conditions may affect not only bacterial physiology but also cell–cell interactions and aggregation. In the oral cavity, microbial ecology is of particular interest in space medicine, as long-duration spaceflight has been associated with alterations in the oral microbiota, immune dysregulation, and an increased risk of oral inflammatory diseases [6,7]. However, whether the effects of SMG on bacterial aggregation are shared among different oral bacterial species or represent species-specific responses remains poorly understood.

Oral biofilm formation is a highly structured ecological process involving sequential colonization by diverse bacterial species with distinct physiological and ecological characteristics [8]. Early colonizers such as *Streptococcus sanguinis*, a Gram-positive facultative anaerobe, mediate initial adhesion to oral surfaces [8]. *S. mutans*, a cariogenic Gram-positive facultative anaerobe, contributes to mature biofilm formation through extracellular polysaccharide production [9]. *Streptococcus salivarius* is a predominant Gram-positive commensal bacterium commonly detected on oral mucosal surfaces and in saliva [10]. Opportunistic pathogens such as *Staphylococcus aureus*, a Gram-positive facultative anaerobe that can transiently colonize the oral cavity, may also participate in oral microbial communities [11]. In contrast, *Porphyromonas gingivalis* is a Gram-negative obligate anaerobe and a major periodontal pathogen that acts as a late colonizer requiring both surface attachment and interspecies interactions for stable biofilm development [8,12].

Notably, *P. gingivalis* expresses two genetically distinct type V fimbriae, FimA fimbriae and Mfa1 fimbriae, which are involved in adhesion, biofilm development, and interbacterial interactions [13,14]. Given their roles in bacterial adhesion and interbacterial interactions, these fimbrial structures may also contribute to aggregate formation under altered environmental conditions. However, whether FimA and Mfa1 contribute to aggregation under SMG remains unclear. The inclusion of these phylogenetically and physiologically distinct oral bacteria therefore provides an opportunity to determine whether the aggregation response observed under SMG is a common feature among oral bacteria or a species-specific phenomenon.

To address this knowledge gap, we investigated the effects of SMG on the growth and aggregation of representative oral bacteria using a clinostat-based system. Aggregation assays of oral streptococci were initially performed in sucrose-containing medium, consistent with previous studies evaluating streptococcal aggregation and biofilm formation. Because previous work with *S. mutans* under SMG was performed in sucrose-containing biofilm medium [5], we further examined the contribution of sucrose to SMG-induced aggregation by comparing cultures grown in the presence and absence of sucrose. In addition, we evaluated whether fimbrial systems in *P. gingivalis* contribute to aggregation under SMG.

## 2. Materials and Methods

### 2.1. Bacterial Strains and Culture Conditions

The bacterial strains used were *S. aureus* 209P, *S. salivarius* HHT, *S. mutans* XC, *S. sanguinis* 10557, and *P. gingivalis* ATCC 33277 together with its isogenic mutant strains JI-1 (Δ*fimA*) [15], SMF (Δ*mfa1*) [16], and SMF-fimA (Δ*fimA* Δ*mfa1*) [17] (Table 1). All strains were revived from frozen stocks and precultured to the stationary phase before each experiment. Facultative anaerobes were cultured in Brain Heart Infusion (BHI) broth at 37 °C. For streptococci, aggregation assays were initially performed in BHI supplemented with 5% (*w*/*v*) sucrose, as commonly used in studies of streptococcal aggregation and biofilm formation [18]. Additional experiments were conducted in sucrose-free BHI to evaluate the contribution of sucrose to SMG-induced aggregation. *P. gingivalis* strains were cultured anaerobically in supplemented trypticase soy broth (sTSB; TSB supplemented with 5 μg/mL hemin and 1 μg/mL menadione) at 37 °C using an anaerobic culture system (Hirasawa Works Inc., Tokyo, Japan).

### 2.2. SMG Exposure

SMG was generated using a three-dimensional clinostat system (ZEROMO, Space Bio-Laboratories, Hiroshima, Japan). The system was operated under conditions designed to achieve an effective gravitational environment of approximately 10^−3^ G by continuously rotating culture vessels around two independent axes. This multidirectional rotation randomized the direction of the gravity vector relative to the samples, thereby averaging the directional effect of gravity over time. Culture vessels were completely filled with medium to eliminate air bubbles and minimize fluid movement during rotation. Normal gravity (NG) controls were cultured under identical environmental conditions without rotation (1 G static condition).

### 2.3. Growth Measurement

Bacterial growth was monitored by measuring the optical density at 600 nm (OD_600_) every 3 h for 24 h using a UV–visible spectrophotometer (Shimadzu, Kyoto, Japan). Growth measurements of the streptococci were performed in BHI supplemented with 5% (*w*/*v*) sucrose, corresponding to the conditions used in the initial aggregation assays. Each experimental condition was evaluated using three independent cultures.

### 2.4. Aggregation Analysis

Cell aggregation was evaluated by phase-contrast microscopy after incubation under NG or SMG conditions. Digital images were analyzed using ImageJ V1.54 software (National Institutes of Health, Bethesda, MD, USA) to quantify aggregate number, total aggregate area, and Feret’s diameter [19]. Owing to uneven background illumination and indistinct aggregate boundaries in side-view images of the culture flasks, automated segmentation was unreliable. Therefore, aggregate boundaries were manually traced for quantitative analysis. The same cultures grown under sucrose-supplemented conditions were also subjected to Gram staining to examine bacterial morphology and aggregate structure by optical microscopy.

### 2.5. Statistical Analysis

Data are presented as the mean ± standard deviation (SD). Comparisons were performed only between two predefined groups. Statistical comparisons were conducted using an unpaired Student’s *t*-test. A *p* value of <0.05 was considered statistically significant.

## 3. Results

### 3.1. Growth Rate Under SMG

**Figure 1:**
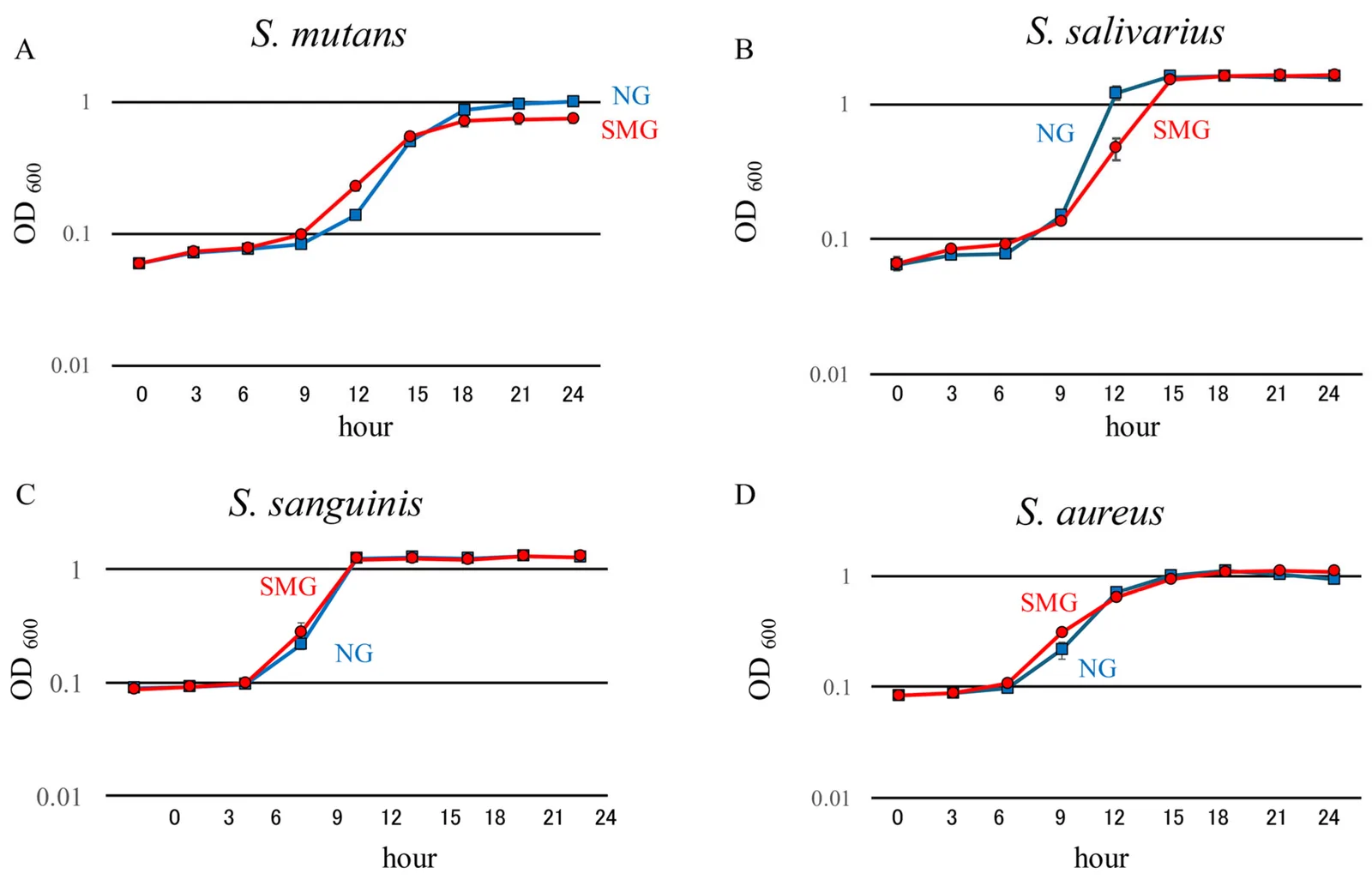
**Growth curves of oral bacteria under normal gravity (NG) and simulated microgravity (SMG).** Growth curves of *Streptococcus mutans*, *Streptococcus salivarius*, *Streptococcus sanguinis* and *Staphylococcus aureus* cultured under NG and SMG conditions. Bacterial growth was monitored by measuring OD_600_ every 3 h, with 24 h as the final time point. Each data point represents the mean of three independent experiments, and error bars indicate the standard deviation (SD). Panels A–D correspond to *S. mutans*, *S. salivarius*, *S. sanguinis* and *S. aureus*, respectively.

Growth rates under SMG and NG were assessed by OD_600_ measurements at 3-h intervals over 24 h (Figure 1). Overall, all bacterial species exhibited similar growth profiles under the two conditions. However, species-specific differences were observed. In *S. mutans*, the onset of the exponential growth phase occurred earlier under SMG than under NG, whereas the final OD_600_ in the stationary phase was lower under SMG than under NG. In contrast, the onset of the exponential growth phase in *S. salivarius* was delayed under SMG compared with NG. No appreciable differences in growth kinetics were observed between SMG and NG for *S. sanguinis* or *S. aureus*.

### 3.2. SMG Induces Species-Dependent Aggregation

**Figure 2:**
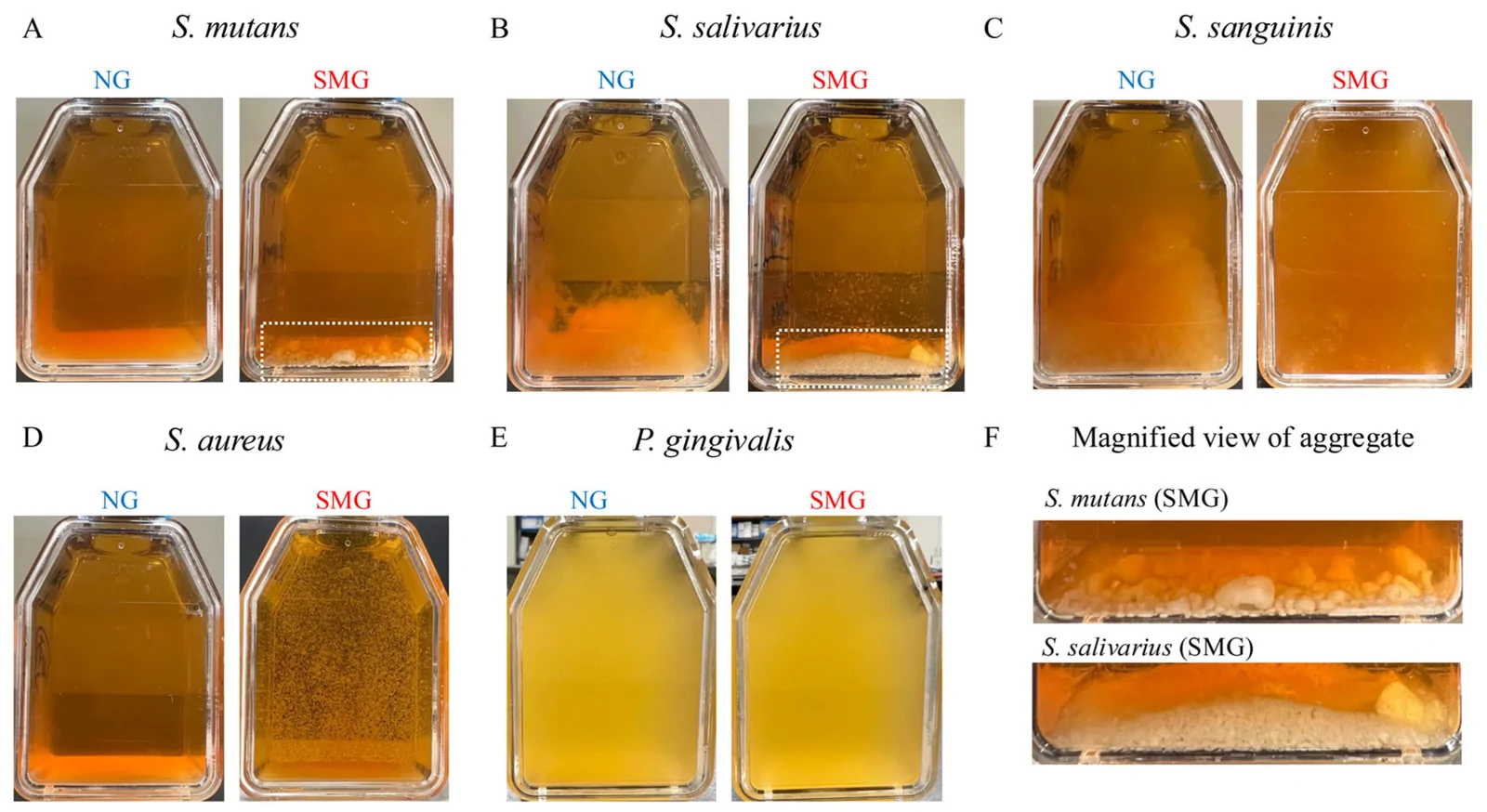
**Representative images of bacterial aggregation under normal gravity (NG).** Side views of vertically positioned culture flasks (12.5 cm^2^, 25-mL capacity, Falcon^®^, Corning Inc., Corning, NY, USA, #353107) containing (**A**) *Streptococcus mutans*, (**B**) *Streptococcus salivarius*, (**C**) *Streptococcus sanguinis*, (**D**) *Staphylococcus aureus* and (**E**) *Porphyromonas gingivalis*. The dashed boxes indicate the regions enlarged in panel (**F**).

Aggregation assays were initially performed under sucrose-supplemented conditions for the streptococcal species. Under SMG, visible sedimented aggregates were observed after incubation, and the extent of aggregate formation differed markedly among bacterial species (Figure 2A–E). Large, compact aggregates were readily observed in *S. mutans* and *S. salivarius*, whereas little or no visible aggregate formation was detected in *S. aureus, S. sanguinis*, or *P. gingivalis*.

**Figure 3:**
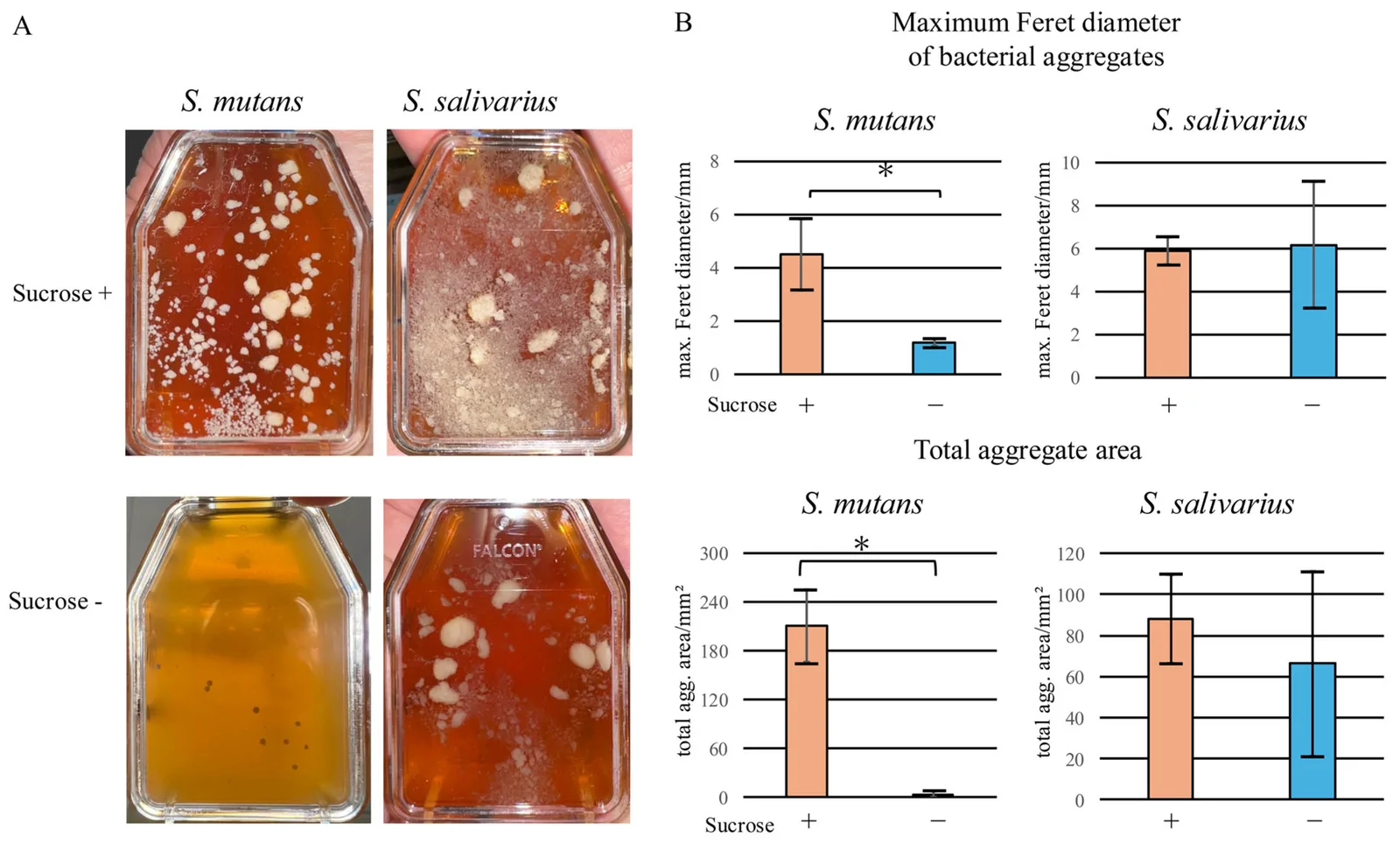
**Effect of sucrose on bacterial aggregation under simulated microgravity (SMG).** (**A**) Representative bottom-view images of horizontally positioned culture flasks under SMG. (**B**) Quantification of bacterial aggregation based on maximum Feret diameter (upper panels) and total aggregate area (lower panels) using ImageJ (n = 3). Left panels, *Streptococcus mutans*; right panels, *Streptococcus salivarius*. An asterisk (*) indicates a statistically significant difference (*p* < 0.05).

Quantitative image analysis confirmed these findings (Figure 3, Table 2). Under sucrose-supplemented SMG conditions, the total aggregate area and maximum Feret’s diameter reached 210.7 mm^2^ and 4.5 mm in *S. mutans*, and 88.3 mm^2^ and 5.9 mm in *S. salivarius*, respectively, indicating robust aggregate formation. The number of aggregates was 96.7 ± 67.4 in *S. mutans* and 12.3 ± 5.5 in *S. salivarius*. In contrast, only minimal or no aggregate formation was detected in *S. aureus*, *S. sanguinis*, and *P. gingivalis*.

### 3.3. Effect of Sucrose on SMG-Induced Aggregation in Streptococci

To further investigate the role of sucrose in SMG-induced aggregation, aggregation assays were repeated in the absence of sucrose.

In *S. mutans*, aggregate formation was markedly reduced under sucrose-free conditions, with only small and sparse aggregates remaining (Figure 3A). Quantitative image analysis demonstrated significant reductions in maximum Feret’s diameter and total aggregate area compared with sucrose-supplemented conditions (*p* < 0.05; Figure 3B).

In contrast, *S. salivarius* retained the ability to form aggregates in the absence of sucrose (Figure 3A). Although number of aggregates and total aggregate area tended to decrease under sucrose-free conditions, these differences were not statistically significant compared with sucrose-supplemented cultures (Figure 3B, Table 2).

### 3.4. Gram Staining Analysis of SMG-Induced Aggregates

**Figure 4:**
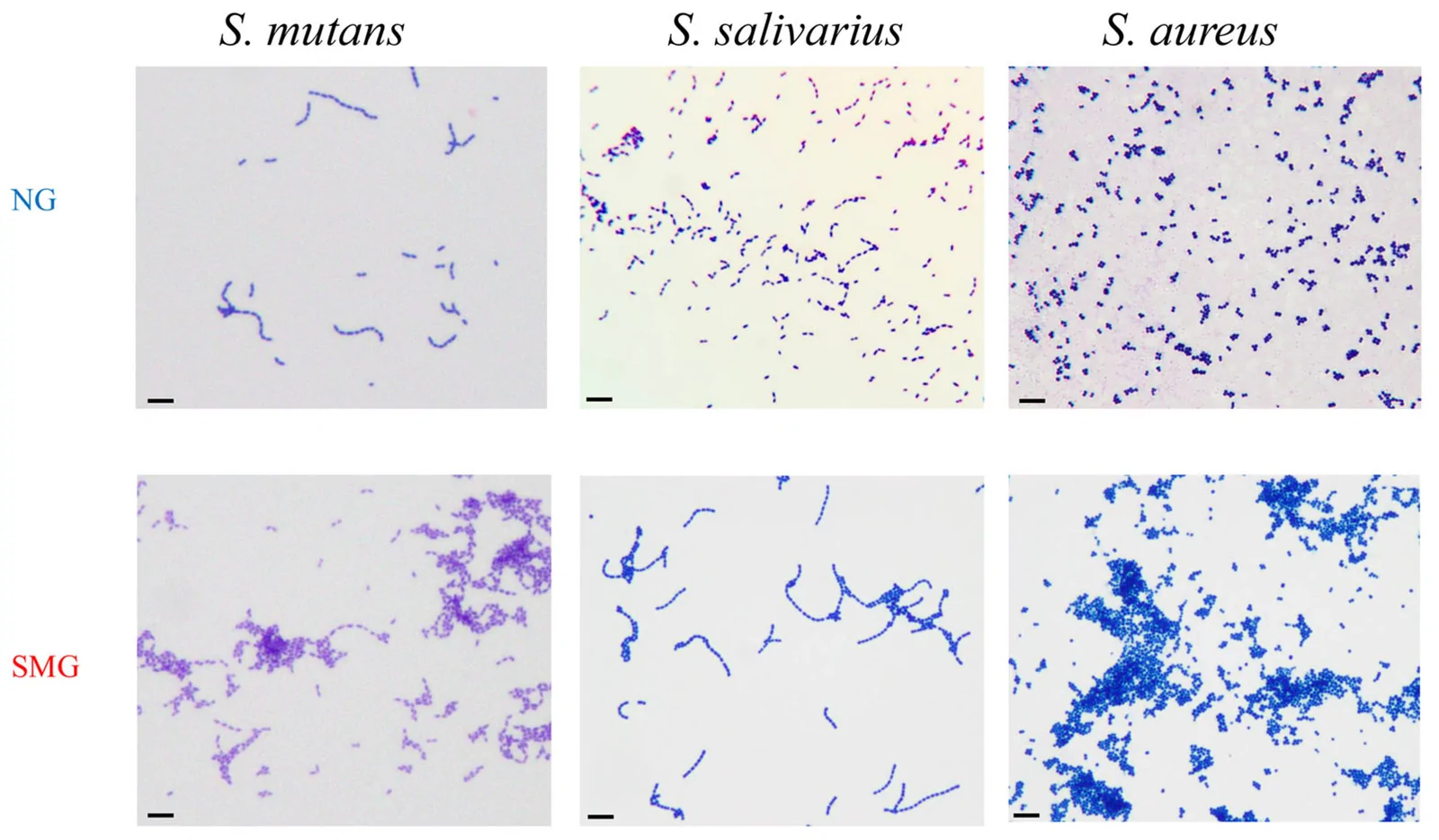
Gram-stained images of bacterial aggregates under normal gravity (NG) and simulated microgravity (SMG) in sucrose-supplemented medium. Representative images of bacterial aggregates formed after incubation under the conditions used for the aggregation assay shown in Figure 2. Scale bars represent 5 μm.

Gram staining analysis revealed that the characteristic cellular arrangements of each species were preserved under both NG and SMG conditions (Figure 4). *S. aureus* retained its typical grape-like clusters, whereas the streptococcal species maintained their chain-like morphology. Under SMG, *S. aureus* tended to form larger grape-like clusters, while *S. mutans* and *S. salivarius* exhibited a tendency toward longer chains. Although these changes did not alter the characteristic cellular arrangement of each species, they suggest that SMG may promote the formation of larger multicellular structures while preserving the inherent morphology of each bacterium.

### 3.5. Aggregation Response of P. Gingivalis

No apparent aggregation was observed in *P. gingivalis* under either NG or SMG conditions (Figure 2E). Furthermore, deletion of *fimA*, *mfa1*, or both genes did not alter the aggregation phenotype under either condition (Figure S1), indicating that these fimbrial systems do not contribute detectably to aggregation under the experimental conditions used in this study.

## 4. Discussion

This study demonstrates that SMG induces species-dependent aggregation among representative oral bacteria. Under the initial experimental conditions using sucrose-supplemented medium for streptococci, pronounced aggregation was observed in *S. mutans* and *S. salivarius*, whereas *S. aureus*, *S. sanguinis*, and *P. gingivalis* exhibited little or no detectable aggregate formation. Growth analysis demonstrated that SMG did not markedly alter overall bacterial proliferation. Although minor differences in growth rate were observed, *S. mutans* exhibited an earlier onset of the exponential growth phase under SMG than under NG, whereas *S. salivarius* showed a delayed onset under SMG. In addition, the final OD_600_ of *S. mutans* during the stationary phase was slightly lower under SMG than under NG. Nevertheless, the overall growth profiles remained comparable between the two gravity conditions. Likewise, no noticeable differences in growth rate were detected for *S. sanguinis* or *S. aureus*. These findings indicate that the enhanced aggregation observed under SMG cannot be explained simply by increased bacterial growth but rather reflects changes in bacterial interactions and aggregate formation. SMG may facilitate an early step of biofilm development by enhancing bacterial aggregation. Bacterial aggregation can increase cell–cell contact and promote the formation of multicellular clusters, which may subsequently enhance the ability of bacterial cells to interact with and attach to surfaces. Such initial aggregation may therefore contribute to the establishment and maturation of biofilms by increasing local cell density and facilitating the accumulation of extracellular matrix components [20]. This interpretation is consistent with previous reports demonstrating that simulated or real microgravity alters bacterial cell–cell interactions, extracellular matrix production, and biofilm architecture [1,5,21]. Although the present study did not directly examine surface adhesion or biofilm formation, the observed species-dependent aggregation under SMG may represent an early microbial event that influences these subsequent processes.

Beyond oral bacteria, both SMG and spaceflight have been shown to alter bacterial physiology in several clinically important pathogens. SMG enhances the virulence of *Salmonella enterica* serovar Typhimurium, whereas spaceflight induces global Hfq-dependent transcriptional responses in *Salmonella* and *Pseudomonas aeruginosa*, together with changes in bacterial adaptation and virulence [22,23,24]. Likewise, microgravity-associated environments influence the physiology and virulence-related phenotypes of *Staphylococcus aureus* [25]. Collectively, these findings, together with the present results, suggest that reduced-shear environments broadly modulate bacterial physiology in a species-dependent manner, affecting multicellular behavior and host interactions across diverse bacterial species.

To further clarify the mechanisms underlying SMG-induced aggregation, the role of sucrose was examined in the two aggregating streptococcal species. Although both *S. mutans* and *S. salivarius* exhibited enhanced aggregation under SMG, their responses to sucrose removal differed substantially. Aggregation in *S. mutans* was markedly reduced under sucrose-free conditions, whereas *S. salivarius* retained the ability to form aggregates despite a slight reduction. These findings are consistent with the established role of glucan-mediated adhesion in *S. mutans,* where glucosyltransferase-derived extracellular glucans are essential for sucrose-dependent cell–cell adhesion and biofilm formation [26]. In contrast, *S. salivarius* possesses intrinsic autoaggregation properties mediated by cell-surface proteins and other adhesins, which may account for its ability to aggregate independently of sucrose [27].

Consistent with the Gram staining observations, SMG promoted the formation of larger grape-like clusters in *S. aureus* and longer streptococcal chains while preserving the characteristic cellular arrangement of each species, suggesting that SMG enhances multicellular organization rather than altering bacterial morphology.

The mechanism underlying SMG-enhanced aggregation is likely related to the unique fluid dynamic conditions generated under SMG. Suppression of sedimentation and convective flow creates a low-shear environment that reduces the physical disruption of developing aggregates [1]. Under these conditions, extracellular polymeric substances (EPS) may accumulate more efficiently around bacterial cells and remain associated with the cell surface, thereby facilitating stable cell–cell interactions [21]. In addition, reduced fluid mixing may prolong contact between neighboring cells, promoting intercellular bridging and aggregate maturation. These physical effects may explain why aggregation increased despite only minimal differences in bacterial growth [5].

In addition to these physical effects, previous studies have shown that SMG alters global gene expression in *S. mutans*, including genes involved in stress responses, carbohydrate metabolism, and biofilm formation [5]. These findings suggest that SMG influences both the physical properties of the culture environment and bacterial physiology, thereby promoting bacterial adhesion. The physical effects of reduced shear may be further reinforced by physiological adaptations that enhance bacterial adhesion. The persistence of aggregation in *S. salivarius* under sucrose-free conditions suggests the involvement of sucrose-independent adhesion mechanisms. *S. salivarius* is a predominant commensal bacterium of the oral mucosa and saliva and possesses intrinsic autoaggregation properties that are thought to be mediated by cell-surface proteins and other adhesive factors [28]. The present findings therefore suggest that SMG may enhance multiple forms of bacterial adhesion, with the predominant mechanism differing among species. In contrast, *P. gingivalis* did not exhibit detectable aggregation under SMG, and disruption of *fimA*, *mfa1*, or both genes did not alter this phenotype. These findings suggest that FimA and Mfa1 fimbriae are not sufficient to promote SMG-induced aggregation, indicating that the aggregation-enhancing effects of SMG primarily involve mechanisms other than fimbria-dependent adhesion.

Several limitations of this study should be acknowledged. First, the experiments were performed using an SMG model, which reproduces key aspects of the low-shear environment encountered during spaceflight but cannot fully replicate true microgravity. Second, although the results suggest that species-specific adhesion mechanisms contribute to SMG-induced aggregation, neither gene expression nor EPS production was directly evaluated. Consequently, the molecular basis of the observed responses remains unclear. Third, the present study used single-species cultures, whereas oral biofilms in vivo consist of complex multispecies communities [8]. Future studies combining transcriptomic analyses, quantitative EPS measurements, and polymicrobial biofilm models will provide further insight into how microgravity influences oral microbial ecology.

The present findings suggest that SMG may facilitate the initial stages of dental plaque development by promoting bacterial aggregation in selected oral streptococci. Although aggregation alone does not represent mature biofilm formation, enhanced cell–cell adhesion under microgravity may create favorable conditions for subsequent plaque maturation. Future studies using multispecies biofilm models will be necessary to determine whether these aggregation-promoting effects contribute to accelerated dental plaque development.

## 5. Conclusions

SMG promotes aggregation of selected oral bacteria through species-specific mechanisms without markedly affecting bacterial growth. These findings suggest that microgravity may alter the organization of oral microbial communities by enhancing specific intercellular adhesion pathways.

## Figures and Tables

**Table 1:** Bacterial strains and culture media used in this study.

Species	Strain	Characteristics	Culture Condition	Source
*Staphylococcus aureus*	209P	Opportunistic pathogen; Gram-positive facultative anaerobe	BHI + 5% sucrose	Laboratory stock
*Streptococcus mutans*	XC	Cariogenic bacterium; Gram-positive facultative anaerobe	BHI ± 5% sucrose	Laboratory stock
*Streptococcus salivarius*	HHT	Oral commensal; Gram-positive facultative anaerobe	BHI ± 5% sucrose	Laboratory stock
*Streptococcus sanguinis*	10557	Early colonizer; Gram-positive facultative anaerobe	BHI + 5% sucrose	Laboratory stock
*Porphyromonas gingivalis*	ATCC 33277	Periodontal pathogen, Wild-type; Gram-negative obligate anaerobe	sTSB	Laboratory stock
	JI-1	ATCC 33277-derived Δ*fimA* mutant		[15]
	SMF	ATCC 33277-derived Δ*mfa1* mutant		[16]
	SMF-fim	ATCC 33277-derived Δ*fimA* Δ*mfa1* double mutant		[17]

BHI, Brain Heart Infusion broth; sTSB, supplemented trypticase soy broth (TSB supplemented with 5 μg/mL hemin and 1 μg/mL menadione).

**Table 2:** Quantitative analysis of bacterial aggregation under sucrose-supplemented and sucrose-free conditions.

Species	Sucrose	Number of Aggregates	Total Aggregate Area (mm^2^)	Mean Feret’s Diameter (mm)	Maximum Feret’s Diameter (mm)
*S. mutans*	+	96.7 ± 67.4	210.7 ± 44.6	2.2 ± 0.6	4.5 ± 1.3
	−	4.0 ± 4.4	3.4 ± 3.9	1.1 ± 0.1	1.2 ± 0.1
*S. salivarius*	+	12.3 ± 5.5	88.3 ± 21.5	3.5 ± 0.4	5.9 ± 0.6
	−	7.0 ± 5.2	66.3 ± 45.2	5.4 ± 3.3	6.2 ± 3.0
*S. aureus*	+	ND	ND	ND	ND
*S. sanguinis*	+	ND	ND	ND	ND
*P. gingivalis*	−	ND	ND	ND	ND

ND, not detected (no measurable aggregates under the analysis conditions). Values are expressed as mean ± standard deviation from three independent experiments (n = 3).

## Data Availability

The data presented in this study are available from the corresponding author upon reasonable request.

## Supplementary Materials

The following supporting information can be downloaded at: https://www.mdpi.com/article/10.3390/life16091402/s1, Figure S1: Representative images of *Porphyromonas gingivalis* cultures under normal gravity (NG) and simulated microgravity (SMG). Side views of verticallypositioned culture flasks showing the wild-type and fimbrial mutant strains (JI-1, SMF1, and SMF-fim) cultured under NG and SMG conditions. No apparentbacterial aggregation was observed in any strain under either condition.
