# Supplementary material for: Species-Dependent Aggregation of Oral Bacteria Under Simulated Microgravity
Source: Life (Basel). 2026 Aug 25;16(9):1402. doi: 10.3390/life16091402 (PMC13608611; doi:10.3390/life16091402)
Supplement: Supplementary file 1 [file life-16-01402-s001.zip › life-4500988-supplementary.pdf]

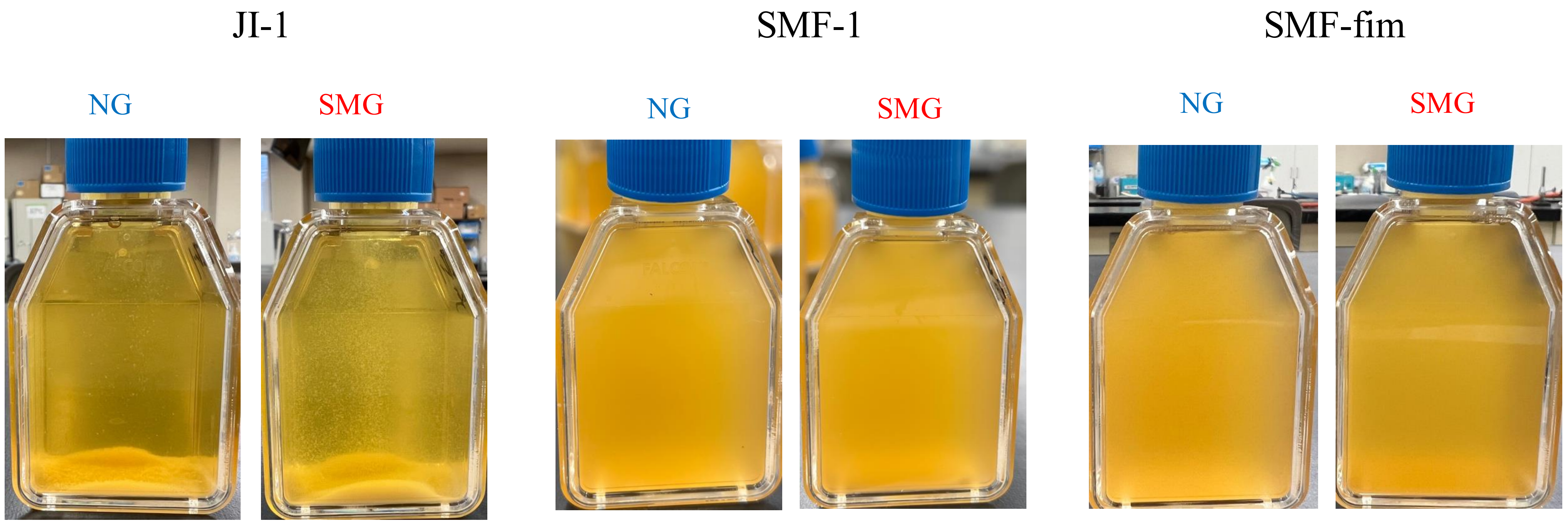

Supplementary figure S1. Representative images of *Porphyromonas gingivalis* cultures under normal gravity (NG) and simulated microgravity (SMG). Side views of vertically positioned culture flasks showing the wild-type and fimbrial mutant strains (JI-1, SMF1, and SMF-fim) cultured under NG and SMG conditions. No apparent bacterial aggregation was observed in any strain under either condition.
